# Therapeutic Notes and Miscellany

**Published:** 1891-01

**Authors:** 


### Therapeutic Notes and Miscellany.

---

THE FIRST THERAPEUTIC MEASURES IN APOPLEXY.—The treatment of apoplexy is apparently so self-evident, and has for decades remained unchanged, that at first it seems preposterous to make any changes or alteration whatever.

Doubtless the experience of H. has often occurred to many physicians in their regular practice. The doctor is called to see a case of extensive apoplexy, and elicits this history: The patient has suffered a light attack of paralysis; he complains of vertigo and a sensation of weight and immobility in both the upper and lower extremities; there is distortion of the facial muscles, and speech is impaired. The patient has been undressed and placed upon a bed; scarcely has this been done, when a second, more profound attack occurs.

This and similar scenes constantly recur in practice; invariably the profound attack occurs shortly after the patient has been placed upon the bed in the horizontal position. This is done again and again, despite the fact that this position conduces to the cerebral hemorrhage, defeats the very object which it is desired to accomplish, *i. e.*, prevention of recurrent profuse hemorrhage.

After these experiences, it is absolutely necessary that after the first, the mild attack of cerebral hemorrhage, the patient shall be maintained in the sitting-erect position for a long time; so long, in fact, as the patient's condition will permit. Meanwhile, ice to the head, hot mustard foot-baths, rapidly acting hydragogue cathartics; and in selected cases, leeches, will be the prophylactic measures which in conjunction with position, as above described, will often prevent a second attack.—*Dr. Heidenhain, Berlin Klin. Wochen.*

---

WHEN urine contains an unusual percentage of carbonic acid gas, it effervesces on the addition of nitric acid. This effervescence often occurs in the case of persons undergoing a course of aerated mineral waters, or habitual indulgers in the products of the soda fountain.

Pilocarpine in Dryness of the Tongue.—Extreme dryness of the tongue is, under any circumstances, a very distressing symptom, and one which does not readily yield to treatment whilst the concomitant cause remains in operation. The sucking of ice or sipping of bland fluids gives but temporary and inadequate relief, and the same may be said of glycerine employed as a paint. In this condition I have successfully used pilocarpine, grain 1-20th or grain 1-10th, in the form of a gelatine lamel, allowed to dissolve on the tongue, previously moistened with a sip of water. I find this small dose quickly establishes a moderate flow of saliva, which persists for at least twenty-four hours, and is unaccompanied by excessive perspiration. The altered state of the mouth is often described by the patient as being delightful. I send this with the hope that others may share the satisfaction I have experienced, if they have not already done so, in this use of pilocarpine. It is scarcely necessary to add that we must exercise due caution in the use of so potent a remedy.—J. G. Blackman, M. D., *Brit. Med. Journal.*

---

As Aristol is now claiming the attention of the medical world, we publish some of its modes of administration, taken from *The Bulletin Général de Thérapeutique.*

1.—Ethereal Solution:

Aristol .................................... ½ ounce.

Ether ...................................... 5 ounces.

2.—With Collodion:

Aristol .................................... 1 part.
Collodion .................................. 9 parts.

3.—Ointment:

Aristol .................................... 1 part.
Olive oil .................................. 20 parts.
Lanoline ................................... 70 parts.

4.—Suppository:

Aristol .................................... 16 grains.
Cocoa butter ............................... q. s.
One suppository ...........................

---

Urine is ammoniacal in many bladder diseases, in grave cases of typhus, in affections of the spinal cord, and in the second stage of acute exudations.

Preparations of Digitalis.—Continental physicians have recently been discussing the efficacy of preparations of digitalis, and one of them focused the general ideas when he said that he preferred the cold infusion on account of its supposed greater diuretic activity. It is prepared by taking from 0.25 to 0.40 gramme of powdered digitalis leaves and adding 300 grammes of cold water. This should be allowed to macerate for twelve hours, and then filtered. It may be sweetened with syrup. When it is desired to obtain a powerful diuretic action, it is preferable to administer this quantity in two or three doses. There is nothing very new about this, but it is well to print it as a reminder that preparations of digitalis containing alcohol are often inert.

---

If a sound, bougie, or catheter is arrested at *less* than six inches from the meatus, the obstruction is caused by a stricture, in all probability ; if it is not stopped until it has passed *more* than six inches, the blame is due to enlarged prostate. Stricture never exists here, but is frequently found at the juncture of the membranous portion with the bulb. Yet the prostate may be enlarged *without* causing any hindrance to the passage of an instrument, especially when the enlargement only involves the third lobe, or when the employed instrument is of sufficient length and suitable curve.

In treating strictures, there is never any necessity to pass the instrument further than nine inches from the point ; this can conveniently be marked on the bougie, and will show that the bladder has been entered. Mischief may be done by forcing it onward against the lining mucous membrane, while no object is served.—*Medical World.*

---

Gum-Lancing in Children.— Dr. Forchheimer says of gum-lancing : 1. It is useless (*a*) as far as giving relief to symptoms ; (*b*) as far as facilitating or hastening teeth. 2. It is useful only as blood-letting, and ought not to be used as such. 3. It is harmful (*a*) in producing local trouble ; (*b*) in producing general disturbance on account of hemorrhage ; (*c*) in having established a method which is too general to do specific good, and too specific for universal use. 4. It is to be used only as a surgical procedure to give relief to surgical accidents.

